# Questions Ophthalmology Trainees Ask About Ocular Syphilis: A Question-Driven Narrative Review

**DOI:** 10.7759/cureus.115917

**Published:** 2026-09-07

**Authors:** Francesco Pichi

**Affiliations:** 1 Department of Ophthalmology, University of Toronto, Toronto, CAN

**Keywords:** acute syphilitic posterior placoid chorioretinitis, medical education, multimodal imaging, neurosyphilis, ocular syphilis, syphilitic uveitis

## Abstract

Syphilis is undergoing a global resurgence, and ocular syphilis, although uncommon, is a sight-threatening manifestation that poses recurrent diagnostic and management difficulties for clinicians in training. The objective of this review was to identify the most frequent questions regarding ocular syphilis raised by ophthalmology residents and to answer them by evaluating the current literature. This was a narrative review informed by a question-based educational needs assessment. Between 2024 and 2025, a standardized invitation was distributed to ophthalmology residents through several international uveitis societies. Questions were submitted voluntarily and anonymously, and overlapping items were consolidated and grouped into thematic domains while preserving their original intent. For each domain, a focused appraisal of the peer-reviewed literature, including the 2021 Centers for Disease Control and Prevention (CDC) sexually transmitted infection treatment guidelines and the 2024 CDC laboratory recommendations for syphilis testing, was synthesized into a direct answer. Of the 53 residents who responded, 18 submitted questions concerning ocular syphilis. These 18 respondents submitted 26 syphilis-related questions, which were consolidated into 15 questions across seven domains: epidemiology, staging, laboratory diagnosis, imaging, systemic evaluation, treatment, and follow-up. They concentrated on serologic interpretation, systemic evaluation, and the logistics of treatment rather than on the clinical recognition of uveitis, clustering at the interface between ophthalmology and infectious disease. Several recurring premises, including the assumptions that ocular syphilis corresponds to a single stage, that imaging can establish the diagnosis, and that lumbar puncture is always required, were found to be outdated or oversimplified. Ocular syphilis is best managed with a neurosyphilis treatment regimen: diagnosed through the combined use of treponemal and nontreponemal serologic tests, supported but not defined by characteristic posterior segment imaging patterns, and monitored with serial nontreponemal titers in shared care with infectious disease services. HIV testing should be strongly recommended in every patient. A question-driven format targets the specific uncertainties faced by residents and may serve as a template for educational reviews in other uveitic entities.

## Introduction and background

Syphilis, a sexually or congenitally acquired infection caused by the spirochete Treponema pallidum, has undergone a well-documented global resurgence over the past two decades. The World Health Organization estimated that new cases among adults rose from 7.1 million in 2020 to 8.0 million in 2022, and comparable upward trends have been reported across the United States, Europe, China, and South America [1,2]. Ocular involvement has followed this trajectory. Uveitis accounts for the majority of ocular presentations and may occur at any stage of the disease [3].

Ocular syphilis remains uncommon in absolute terms. A recent analysis of the United States TriNetX database reported, over the period 2013 to 2024, a cumulative incidence of 0.36 and a prevalence of 0.27 cases per 100,000, with affected patients being predominantly older, male, and frequently co-infected with the human immunodeficiency virus (HIV) [4]. Despite its relative rarity, the condition carries a disproportionate clinical importance. Its protean presentation, which may mimic virtually any inflammatory phenotype of the anterior or posterior segment, has earned syphilis the designation of "the great masquerader" [3,5]. A delay in recognition can result in irreversible visual loss, whereas timely antibiotic treatment is associated with visual recovery in a substantial proportion of eyes [6,7].

The diagnostic and therapeutic pathway is less straightforward than the treatable nature of the disease might suggest. Serologic interpretation requires familiarity with treponemal and nontreponemal assays, with the choice between testing algorithms, and with pitfalls such as the prozone phenomenon. The decision to perform lumbar puncture, the role of corticosteroids, the management of penicillin allergy, and the schedule for serologic monitoring are recurring points of uncertainty, and the relevant guidance is dispersed across ophthalmic, infectious disease, and public health literature.

To characterize these knowledge gaps, we solicited questions on uveitis from ophthalmology residents internationally. Syphilis emerged as a recurrent topic. The purpose of this review is to answer the questions on ocular syphilis submitted by residents with evidence from the current literature, producing a resource anchored in the genuine uncertainties of clinicians in training rather than a predetermined didactic outline.

## Review

Materials and methods

This was a narrative review informed by a question-based educational needs assessment. Questions were submitted voluntarily and anonymously as part of an educational needs assessment. No identifying or demographic information was collected. Formal institutional review board review was not sought because the activity was conducted as an anonymous educational exercise rather than as research involving identifiable participants.

Between 2024 and 2025, a standardized invitation was distributed by electronic mail to members of several international uveitis societies, including the International Uveitis Study Group (IUSG), the American Uveitis Society (AUS), and the International Ocular Inflammation Society (IOIS). Recipients were ophthalmology residents who were invited to submit questions they had always wanted to ask a uveitis expert, spanning specific conditions, pathogenesis, classification, diagnosis, laboratory testing, imaging, and treatment. No restriction was placed on the number or subject of questions, and no predefined list of topics was imposed. Because the invitation was distributed through society mailing lists, the total number of recipients could not be determined, and no response rate is reported.

The invitation was distributed internationally through society networks spanning Australasia, the Middle East, Africa, Asia, Europe, and the Americas. A total of 53 residents responded with questions covering the breadth of uveitis, of whom 18 submitted questions specifically concerning syphilis. Because respondents were self-selected and the geographic origin of individual submissions was not systematically recorded, the sample should not be regarded as representative of any defined population.

All original questions were retained in the source dataset. Overlapping questions addressing the same underlying concept were combined and lightly edited for presentation while preserving their original intent, so that the questions shown in Table 1 are consolidated and paraphrased rather than verbatim.

**Table 1:** Consolidated questions submitted by the ophthalmology residents, arranged by thematic domain The questions shown are consolidated and paraphrased rather than verbatim; n denotes the number of consolidated questions in each domain (15 in total, from 26 original submissions).

Domain	Consolidated question	n	Key answer
Epidemiology	What are the most current data on syphilis, and how does the burden differ by sex and HIV status?	1	Syphilis is rising; ocular disease is predominantly male; HIV is a common but not universal risk marker.
Staging	Is ocular syphilis a secondary or a tertiary disease?	1	It may occur at any stage; management is stage-independent.
Laboratory diagnosis	What is the difference between the traditional and reverse algorithms, when should each be used, and how are false results and the prozone recognized?	5	Both treponemal and nontreponemal tests are required; algorithm choice is a laboratory decision; exclude a prozone when suspicion is high.
Imaging	Can imaging help provide diagnostic clues?	1	Imaging supports pattern recognition but does not establish the diagnosis.
Systemic evaluation	Should every patient be admitted, and should a lumbar puncture always be performed?	2	Admission depends on the regimen; lumbar puncture is not required in isolated ocular disease with a normal neurologic examination.
Treatment	How is penicillin allergy managed, are there options without admission or penicillin, and should corticosteroids be used and when?	3	Verify the allergy history; desensitization permits standard treatment, while ceftriaxone is an alternative in selected nonpregnant patients; corticosteroids are adjunctive and follow antimicrobial therapy.
Follow-up	Who monitors the titer, and how are an adequate response, serofast state, treatment failure, and reinfection distinguished?	2	Serial nontreponemal titers in shared care; a four-fold fall generally supports an adequate response, a persistent low titer suggests a serofast state, and a four-fold rise should raise concern for treatment failure or reinfection.

The 18 respondents submitted 26 syphilis-related questions, which were consolidated into 15 questions and grouped by content into seven thematic domains: epidemiology; staging; laboratory diagnosis; imaging; systemic evaluation and neurosyphilis; treatment; and follow-up. Questions were considered to address the same underlying concept when they asked about the same clinical decision or the same diagnostic or therapeutic step, even when phrased differently; such questions were merged into a single consolidated item, and the consolidated items were then assigned to the thematic domain matching their primary content. Question consolidation and thematic allocation were performed by a single author without formal assessment of inter-rater agreement, and no predefined consolidation criteria were established in advance. The consolidated questions and the domain in which each was placed are summarized in Table 1.

For each domain, a literature search was performed in PubMed, complemented by Google Scholar for exploratory purposes. Search terms combined "ocular syphilis," "syphilitic uveitis," "neurosyphilis," "serology," "lumbar puncture," "corticosteroids," "ceftriaxone," "doxycycline," "prozone," and "multimodal imaging." English-language guidelines, systematic reviews, cohort studies, and clinically relevant case series were prioritized, and the reference lists of selected articles were screened manually. Searches were last updated on December 31, 2025. Where high-quality evidence was limited, expert consensus and established clinical practice were reported and identified as such. Consensus documents were distinguished by scope, namely the 2021 Centers for Disease Control and Prevention (CDC) sexually transmitted infection treatment guidelines for management and the 2024 CDC laboratory recommendations for syphilis testing for diagnostics.

Results

Of the 53 residents who responded, 18 submitted at least one question concerning syphilis. These respondents submitted 26 syphilis-related questions, which were consolidated into 15 questions across seven domains (Table 1). The consolidated questions were distributed unevenly across the domains: laboratory diagnosis accounted for the largest share (five consolidated questions), followed by treatment (three) and by systemic evaluation and follow-up (two each), while epidemiology, staging, and imaging each yielded a single consolidated question. The questions concentrated on serologic interpretation, systemic evaluation, and the logistics of treatment, whereas none concerned the basic clinical recognition of uveitis. Recurrent items included which serologic test to order and how to interpret discordant results, whether to perform a lumbar puncture, whether to admit the patient, and how to distinguish an adequate response from a serofast state and reinfection. This distribution indicates that the uncertainties cluster at the interface between ophthalmology and infectious disease rather than in the recognition of intraocular inflammation itself. The evidence-based answers to each domain are presented in the sections that follow. Evidence-based answers to trainee questions are given below.

Epidemiology: What are the Most Current Data on Syphilis, and How Does the Burden Differ by Sex and HIV Status?

At the systemic level, syphilis has been in sustained resurgence. The World Health Organization estimated that new cases among adults aged 15 to 49 years rose from 7.1 million in 2020 to 8.0 million in 2022, with the largest increases in the Region of the Americas and the African Region [1]. The Americas alone accounted for approximately 3.37 million new cases in 2022, roughly 42 percent of the global total [8]. In the United States, national surveillance recorded more than 207,000 cases of syphilis across all stages in 2022, the highest number since 1950, including approximately 59,000 cases of primary and secondary syphilis [2].

Sex distribution requires a distinction between systemic and ocular disease. Systemically, the historical male predominance has narrowed: the World Health Organization estimated the 2022 global prevalence of active syphilis at 0.6 percent in both sexes, and United States surveillance has documented a disproportionate rise among women, reflected in the resurgence of congenital syphilis [1,2]. In ocular syphilis, however, a male predominance persists, with 75 to 94 percent of male patients across contemporary series and a large United States database analysis reporting approximately 75 percent [4,6,9]. This difference is commonly attributed to the epidemiology of HIV co-infection and of men who have sex with men (MSM), although referral and testing patterns may also contribute and the association should be interpreted with caution [10,11].

HIV co-infection was the epidemiological point raised most often. It is common but not universal, and reported rates in ocular syphilis vary widely across geography, period, and population, ranging from approximately 25 to 75 percent, with larger series often near one half [3,12,13]. A CDC review of 388 patients across eight jurisdictions reported HIV positivity in 51 percent [12], and a case-control study in British Columbia found HIV in 48.5 percent of ocular syphilis cases, significantly more than among syphilis patients without ocular disease [13]. HIV co-infection is therefore best regarded as a strong risk marker for ocular involvement rather than a prerequisite. The degree of immunosuppression, reflected in the CD4 count, may matter more than serostatus alone [13-15], and because a substantial proportion of patients are found to be HIV-positive only during the ocular workup, HIV testing should be strongly recommended in every case [10,16].

Staging: Is Ocular Syphilis a Secondary or a Tertiary Disease?

The most useful answer reframes the question, because the premise that ocular involvement maps onto a single stage is not supported by the natural history of the infection. Acquired syphilis is divided into primary, secondary, latent, and tertiary stages, and ocular involvement has been documented at each [3,17]. Although intraocular inflammation is frequently reported during secondary or latent infection, no ocular phenotype maps reliably to a single systemic stage, and posterior placoid disease should not be interpreted as evidence of tertiary syphilis [3,18]. Syphilis testing should therefore be included in the evaluation of otherwise unexplained uveitis and other compatible inflammatory ocular presentations, irrespective of apparent stage.

More important for practice is that staging does not determine management. Since 2010, and reaffirmed in subsequent guidance, any ocular manifestation of syphilis is treated with a regimen appropriate for neurosyphilis, regardless of clinical stage [19,20]. Because central nervous system invasion by T. pallidum can occur at any stage, ocular syphilis is managed according to neurosyphilis treatment recommendations even when the systemic stage appears early and cerebrospinal fluid examination is normal [20]. The practically correct answer is therefore that ocular syphilis should be regarded not as secondary or tertiary disease but as a manifestation that requires treatment with a neurosyphilis regimen.

Laboratory Diagnosis: Which Tests Should be Ordered, When is Each Algorithm Used, and How are False Results and the Prozone Recognized?

Serologic testing rests on two complementary categories. Nontreponemal tests (Rapid Plasma Reagin (RPR), Venereal Disease Research Laboratory (VDRL)) detect antibodies to lipoidal antigens; their titers rise with active infection and fall after treatment, making them quantitative and suitable for monitoring. Treponemal tests (Fluorescent Treponemal Antibody Absorption test (FTA-ABS), Treponema Pallidum Particle Agglutination (TP-PA), and automated enzyme and chemiluminescence immunoassays) detect antibodies to treponemal antigens; they confirm exposure but typically remain reactive for life and cannot distinguish active from past, treated infection [21]. In the traditional algorithm a nontreponemal test is performed first and reactive samples are confirmed with a treponemal test; in the reverse algorithm an automated treponemal immunoassay is performed first, reactive samples are reflexed to a quantitative nontreponemal test, and a discordant result is adjudicated by a second, different treponemal assay [21,22].

The choice of algorithm has no single universal answer, which is itself the point residents need. The 2024 CDC laboratory recommendations consider both algorithms acceptable and advise that the choice reflect patient population, test volume, cost, and workflow [21]. The traditional algorithm suits smaller, lower-volume laboratories, while the reverse algorithm improves workflow in higher-volume settings and detects more early primary and late latent infection [22,23]. In the individual patient with unexplained ocular inflammation the recommendation is unambiguous: ensure that testing includes both a treponemal and a quantitative nontreponemal assay, whether ordered separately or performed through a complete reflex algorithm, because reliance on one category risks missing the diagnosis [18,21].

The two categories fail differently. Nontreponemal tests may give biological false-positive results, usually at low titer, in pregnancy, older age, autoimmune disease, and other infections, and may be falsely negative in early primary and in late untreated disease. Treponemal tests are highly sensitive and specific but not immune to false positivity, particularly with other treponemal diseases. Because neither is a gold standard, all results must be correlated with the clinical presentation [21]. Prior treatment affects the two categories differently: treponemal antibodies usually persist for life, except that approximately 15 to 25 percent of patients treated during primary syphilis may serorevert within two to three years, whereas the nontreponemal titer declines after effective treatment and is used to gauge activity and monitor response [21].

The prozone phenomenon deserves attention because it produces a false-negative result in the patients most likely to have active disease. An excess of nontreponemal antibody prevents lattice formation, so undiluted serum is nonreactive despite a high true titer; dilution restores reactivity. It affects nontreponemal but not agglutination-based treponemal tests. In absolute terms it is rare, occurring in fewer than 0.85 percent of specimens in two large referral series, but it is more frequent in primary (4.7 percent) and secondary (1.8 percent) syphilis and in HIV co-infection [21,24,25]. When clinical suspicion for active syphilis remains high despite a nonreactive nontreponemal test, the laboratory should be asked to repeat testing with serial dilutions to exclude a prozone, occasionally to 1:256 or 1:512 [21]. In reverse-sequence screening, by contrast, a discordant treponemal-positive, nontreponemal-negative result is common in previously treated, late, or very early infection and in false positivity, and is adjudicated with a second, different treponemal assay rather than by dilution.

Imaging: Can Imaging Help Provide Diagnostic Clues?

The answer depends on framing. No single imaging finding can unmask syphilis across the full breadth of its presentations, and imaging must never substitute for serology. Approached as pattern recognition in the posterior segment, however, imaging is a useful adjunct: four recurring patterns are characteristic enough that, in the right context, they raise suspicion and prompt confirmatory testing [26,27].

The first is superficial retinal precipitates: creamy-yellow deposits over areas of a diaphanous, ground-glass retinitis, sometimes wedge-shaped, that sit on the retinal surface without involving the underlying retina and migrate over its surface during disease and treatment [28]. Although termed precipitates, they are better understood as focal accumulations of inflammatory cells [29]. They are highly suggestive of syphilis and have occurred in patients not previously known to be infected [28]. The second is multifocal retinitis, in which intraretinal foci are scattered across the posterior pole and have repeatedly been reported after systemic or intravitreal corticosteroids given without anti-treponemal cover [30,31]; the pattern is a practical reminder to exclude syphilis before immunosuppressing undifferentiated posterior uveitis [27,32].

**Figure 1:**
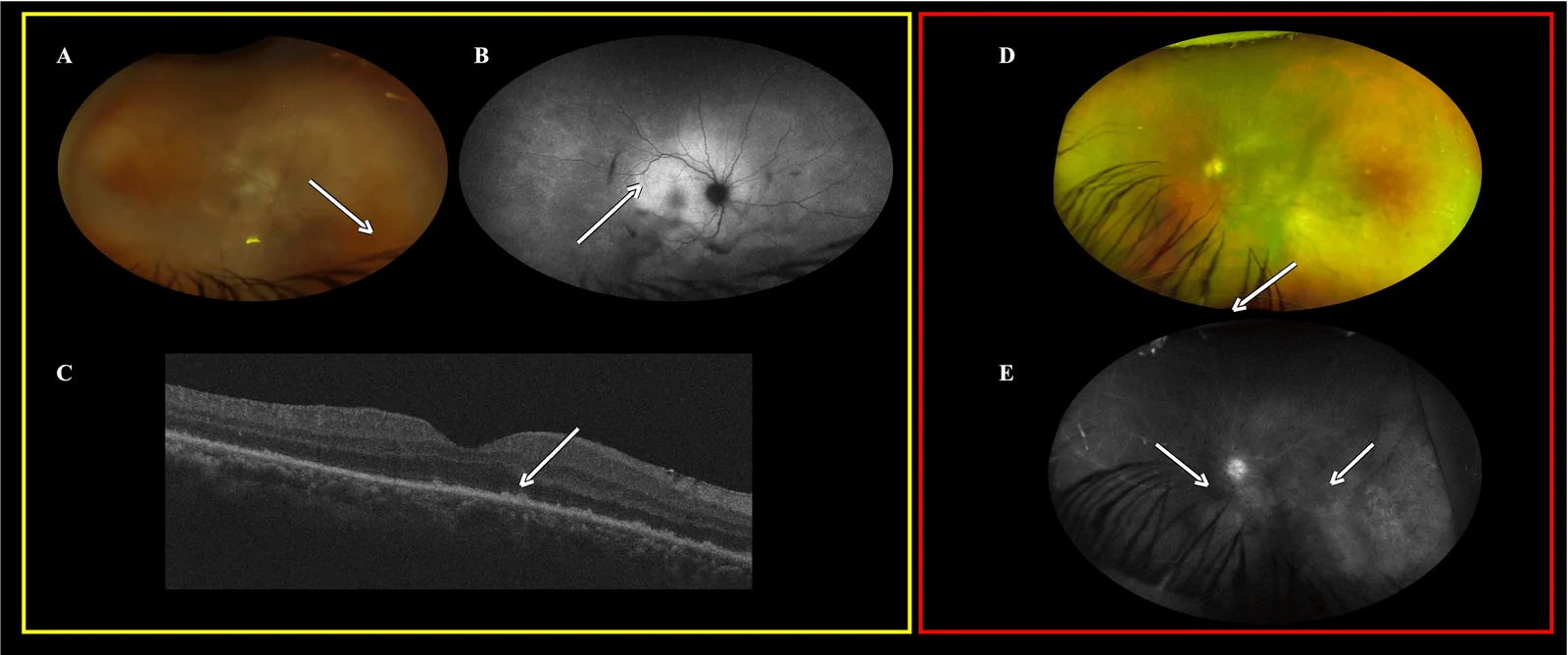
Multimodal imaging of two posterior segment patterns of ocular syphilis (A-C) Acute syphilitic posterior placoid chorioretinitis: (A) widefield color fundus photograph showing a placoid, yellowish outer retinal lesion at the posterior pole; (B) fundus autofluorescence showing hyperautofluorescence of the active lesion; (C) spectral-domain optical coherence tomography showing disruption of the ellipsoid zone with a thickened, granular, hyperreflective retinal pigment epithelium. (D, E) Confluent (triangular) syphilitic retinochoroiditis: (D) widefield color fundus photograph showing a ground-glass, wedge-shaped area of retinochoroiditis; (E) fluorescein angiography showing hyperfluorescence corresponding to the lesion. In each panel, the arrow indicates the principal finding: the placoid macular lesion (A), the corresponding hyperautofluorescence (B), the disrupted, thickened outer retina at the level of the ellipsoid zone and retinal pigment epithelium (C), the confluent ground-glass area of retinochoroiditis (D), and the areas of hyperfluorescence at the optic disc and in the inferotemporal retina (E).

The third is retinochoroiditis, which involves the retina more than the choroid. The confluent form shows a ground-glass, often triangular appearance that is less densely necrotic than herpetic or toxoplasmic retinitis, is frequently accompanied by vasculitis and overlying accumulations, and shows hyperfluorescence of the lesion margin on angiography (Figures 1D, 1E) [27,28].

A denser variant may resemble acute retinal necrosis or toxoplasmosis, underscoring the need for serologic confirmation. The fourth and most distinctive is acute syphilitic posterior placoid chorioretinitis (ASPPC), which is highly characteristic and strongly suggestive of ocular syphilis: one or more large, yellowish, placoid outer retinal lesions at the level of the retinal pigment epithelium (RPE) in the macula or posterior pole [33,34]. On spectral-domain optical coherence tomography (OCT) the hallmarks are an intact external limiting membrane, disruption of the ellipsoid zone, and a thickened, granular, hyperreflective RPE with nodular elevations developing over the first week; transient subretinal fluid and punctate hyperreflective choroidal spots (approximately 31 percent of eyes at presentation) have been described, with substantial structural recovery reported following penicillin treatment [35,36]. Fundus autofluorescence shows hyperautofluorescence, while fluorescein and indocyanine green angiography show early hypofluorescence and late staining (Figures 1A-1C) [27,35].

These imaging findings have also informed pathogenetic hypotheses. The nodular RPE elevations have been hypothesized to reflect altered RPE metabolism arising from regional choroidal occlusive events, possibly related to choroidal vascular inflammation in response to the systemic infection; this interpretation is consistent with angiographic choriocapillaris hypoperfusion and with rapid recovery after treatment, but direct evidence remains limited [5,27]. Overall, imaging is valuable for the clinician who uses it for pattern recognition, with ASPPC the most reliable pattern, rather than as a means of definitive diagnosis. Representative multimodal imaging of two of these patterns is shown in Figure 1.

Systemic Evaluation and Neurosyphilis: Should Every Patient Be Admitted, and Should a Lumbar Puncture Always Be Performed?

Admission is, in practice, a question about the regimen rather than disease severity. Because ocular syphilis is managed with a neurosyphilis regimen, the recommended treatment is aqueous crystalline penicillin G, 18 to 24 million units per day, given as 3 to 4 million units intravenously every four hours or by continuous infusion for 10 to 14 days [19,20]. This schedule has traditionally required hospitalization because frequent intravenous dosing is difficult to deliver as an outpatient, not because the ocular disease demands inpatient monitoring. Admission is therefore a consequence of the chosen regimen, and alternatives compatible with outpatient care may obviate it in selected, reliable patients. Because delayed treatment is associated with irreversible visual loss, therapy should not be deferred to arrange admission when an appropriate outpatient pathway exists.

Lumbar puncture has a clearer, more current answer. Formerly recommended at diagnosis in all cases, since 2021 it is no longer advised for isolated ocular syphilis with reactive serology and a normal neurologic examination [18,19]. The principal reason is straightforward: because ocular syphilis is already treated with a neurosyphilis regimen, the result does not change management in the neurologically normal patient [19,20]. Cerebrospinal fluid examination is indicated when neurologic abnormalities, including relevant cranial nerve dysfunction, are present, and should be considered in suspected treatment failure, particularly when neurologic findings are present or reinfection is not supported by the exposure history. It is not routinely required in isolated ocular syphilis with a confirmed ophthalmic examination and normal neurologic findings, nor in isolated otosyphilis with a normal neurologic examination [19,20]. The older literature reflects the previous practice, in which a high proportion of patients examined routinely had cerebrospinal fluid abnormalities [12,37]; these observations are consistent with frequent, often subclinical, central nervous system involvement and reinforce rather than contradict the current approach. Evaluation is best conducted with an infectious disease specialist.

Treatment: How Is Penicillin Allergy Managed, Are There Options Without Admission or Penicillin, and Should Corticosteroids Be Used?

The first-line regimen is aqueous crystalline penicillin G, 18 to 24 million units per day for 10 to 14 days; where adherence can be ensured, procaine penicillin 2.4 million units intramuscularly once daily with oral probenecid 500 mg four times daily is a CDC-recognized alternative [19,20]. For penicillin allergy, the allergy history should be verified; desensitization allows standard treatment, while ceftriaxone is a possible alternative in selected nonpregnant patients. When desensitization is not feasible, guidance cites limited data supporting ceftriaxone 1 to 2 g daily for 10 to 14 days, noting that cross-reactivity with third-generation cephalosporins is low [19]. A retrospective comparison found no significant difference in effectiveness between ceftriaxone and penicillin G [38]. The Jarisch-Herxheimer reaction is not specific to penicillin and has been reported after ceftriaxone [39].

Treatment without admission and without penicillin overlaps with the admission question. Ceftriaxone, given once daily intramuscularly or intravenously, avoids the four-hourly dosing that drives admission and can be delivered as an outpatient; a case series reported clinical improvement and satisfactory VDRL decline with outpatient intravenous ceftriaxone [40]. Oral doxycycline is not a guideline-endorsed standard regimen for ocular syphilis; recent observational data are hypothesis-generating and may support its use only in exceptional, carefully selected circumstances when recommended parenteral therapy cannot be delivered [41]. These options are best guided by an infectious disease specialist, and logistical difficulty should never delay effective treatment. Pregnancy is a distinct circumstance, because penicillin remains the only established therapy that treats maternal infection and prevents congenital syphilis; a pregnant patient with a reported allergy requires evaluation and desensitization rather than substitution with doxycycline.

Corticosteroids raised two questions: whether and when. There is no firmly established benefit in ocular syphilis, and the CDC does not officially recommend them [19,20], although they are widely used to control inflammation, most commonly topical or systemic [42]. The critical point is sequence rather than dose: corticosteroids should not be started before adequate antimicrobial cover, because immunosuppression without anti-treponemal therapy may worsen the disease [32,43]. This is supported by reports of ocular syphilis misdiagnosed as viral retinitis or acute retinal necrosis and treated with steroids, with progression and permanent visual loss [43], and by multifocal retinitis reported after corticosteroids given without appropriate therapy [27,32]. Some clinicians administer corticosteroids at or shortly after antibiotic initiation when severe inflammation or a Jarisch-Herxheimer reaction is a concern; however, evidence that corticosteroid premedication prevents the reaction or improves visual outcomes is lacking [39]. Steroids should not delay antimicrobial therapy and should generally not be given without effective antimicrobial coverage. Topical corticosteroids and cycloplegics remain appropriate for anterior segment inflammation.

Follow-up: Who Monitors the Titer, and How Are an Adequate Response, Serofast State, Treatment Failure, and Reinfection Distinguished?

Response is judged by the nontreponemal titer, not the treponemal test. An appropriate serologic response is generally defined as a four-fold decline (two dilutions, e.g. 1:32 to 1:8), expected within six to 12 months in early disease and up to 24 months in late or late latent disease [19,21]. A four-fold decline is an important criterion of serologic response rather than a complete definition of clinical success: the rate of decline depends on stage, some patients respond inadequately without demonstrable persistent infection, ocular recovery may not parallel the titer, and low initial titers may not show a measurable four-fold fall. Two technical requirements are essential and are common sources of error: sequential titers should use the same assay and preferably the same laboratory, because RPR titers run slightly higher than VDRL and the two are not directly comparable [21].

The serologic schedule is influenced by stage, HIV status, clinical response, reinfection risk, and baseline titer rather than by ocular involvement alone (Table 2).

**Table 2:** Suggested serological follow-up by clinical context

Clinical context	Suggested serologic follow-up
Early syphilis	6 and 12 months
Latent syphilis	6, 12, and 24 months
HIV co-infection	More frequent, stage-specific follow-up
Suspected relapse or reinfection	Immediate repeat quantitative titer and clinical reassessment

In parallel, the eye requires its own follow-up of intraocular inflammation and structural recovery. Serologic and ocular responses are complementary but not necessarily parallel: visual recovery, control of inflammation, and improvement on imaging may follow a different course from the decline in nontreponemal titers.

Two patterns short of failure are frequently misread. First, complete seroreversion is not universal: a persistent, stable low-level nontreponemal titer may represent a serofast state and does not by itself indicate active infection, and this pattern is more common in patients treated later or with repeated episodes [21]. Notably, only a minority of serofast patients derive benefit from retreatment [44]. Second, a prospective randomized study found that approximately 14 percent of patients with early syphilis had a less than four-fold decline at 12 months despite recommended treatment, with HIV-positive patients more likely to respond inadequately; an incomplete decline warrants closer follow-up rather than automatic re-treatment [21].

The distinction between failure and reinfection rests on the direction and magnitude of change in the titer. A sustained four-fold increase compared with the previous documented nontreponemal titer, measured with the same assay, or recurrence of compatible signs or symptoms, raises concern for reinfection or treatment failure; in a patient whose titer had declined and then rose four-fold from a stable low baseline, reinfection is more likely, particularly with ongoing sexual risk [19,21]. Because the two cannot be distinguished on serology alone, such patients should be re-treated, re-evaluated for HIV, and considered for cerebrospinal fluid analysis [19]. Monitoring is best shared between the ophthalmologist, who follows the ocular inflammation and visual outcome, and an infectious disease or sexual health specialist, who oversees the systemic serologic response, coordinates re-treatment, and manages partner notification and screening for reinfection.

A Practical Pathway for Suspected Ocular Syphilis

**Figure 2:**
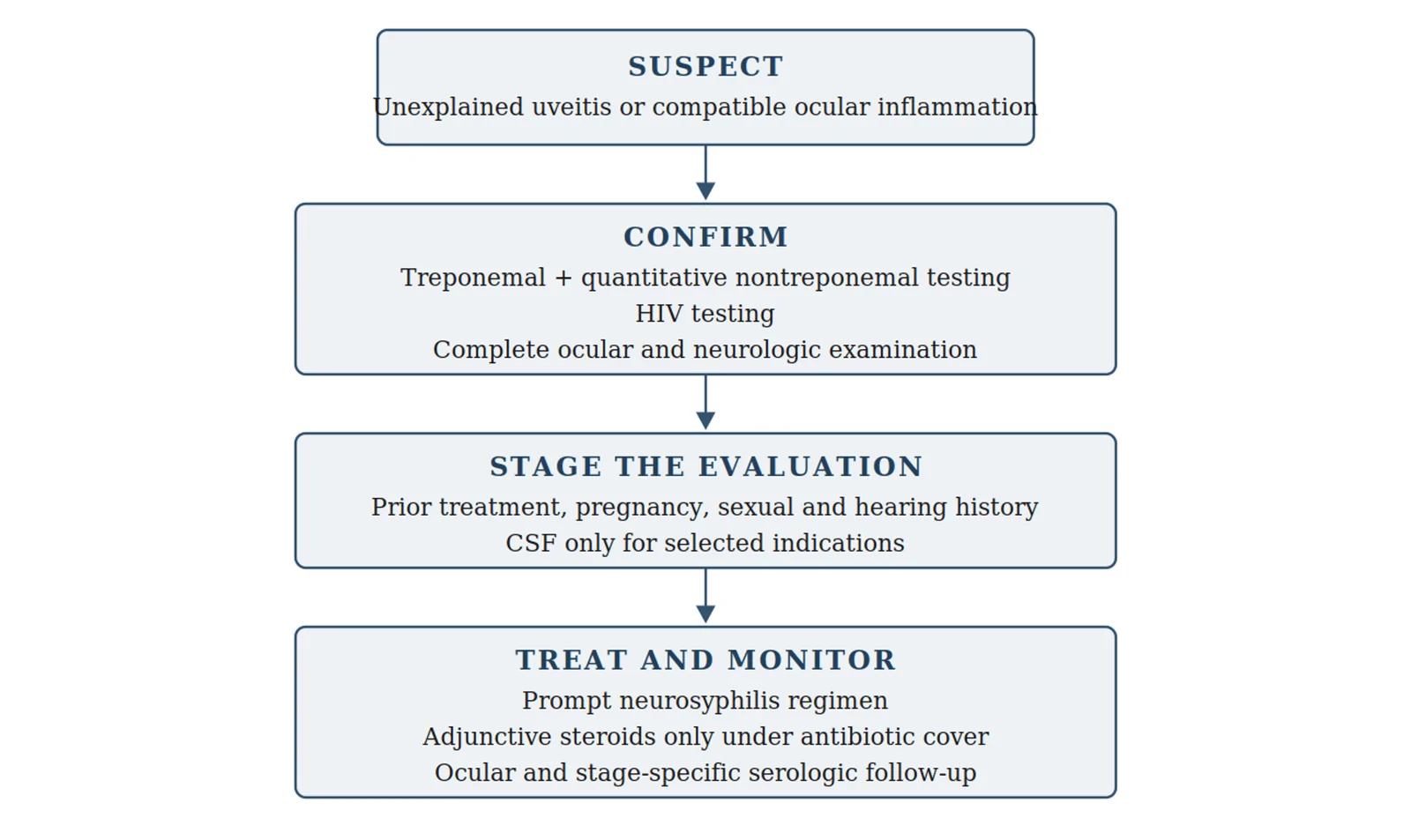
Flowchart consolidating the recurring answers into a four-step pathway usable at the bedside CSF, cerebrospinal fluid.

The pathway in Figure 2 consolidates the recurring answers into a four-step sequence usable at the bedside.

Discussion

This review answered the questions on ocular syphilis submitted by ophthalmology residents internationally and organized them into seven domains. Two observations followed. First, the questions clustered around a small number of recurring uncertainties rather than the full breadth of the topic. Second, a question-driven format revealed where the gap between available evidence and everyday clinical confidence is widest.

The questions were not evenly distributed. They concentrated on serologic interpretation, systemic evaluation, and the logistics of treatment, and comparatively few concerned the recognition of uveitis. Within this self-selected group, this pattern suggests that the responding residents identified intraocular inflammation comfortably but were less certain about the subsequent steps that cross the boundary between ophthalmology and infectious disease. Given the small, self-selected sample, this is best read as an observation from these respondents rather than a general conclusion about ophthalmology trainees. Several premises that residents appeared to hold, that ocular syphilis maps onto a single stage, that imaging can unmask it, or that lumbar puncture is always required, proved outdated or oversimplified; surfacing such misconceptions is a specific value of the question-driven format that a conventional didactic review might not achieve, since the distribution of questions itself identifies where these particular respondents encountered the greatest uncertainty.

A few principles recurred across domains and were the load-bearing concepts a resident most needs to internalize: ocular syphilis is managed with a neurosyphilis regimen; diagnosis rests on complementary serologic tests rather than any single assay or imaging finding, with imaging an adjunct and never a substitute; and corticosteroids, when indicated, should be used only with adequate antimicrobial coverage and should never delay appropriate antibiotic treatment. Consolidating these into the pathway in Figure 2 is intended to make them usable without repeating them in each section.

This review has limitations. It is a narrative review informed by a needs assessment rather than a systematic review, and the literature was appraised in a focused manner; publication bias and the predominance of retrospective series and case reports limit the strength of the evidence, particularly for corticosteroids and non-penicillin regimens. Respondents were self-selected, the total number of invited recipients and the geographic origin of individual submissions were not captured, and no response rate can be calculated; the sample is therefore not representative of any defined population. Question consolidation and thematic allocation were performed by a single author without formal assessment of inter-rater agreement. The number of syphilis-specific questions was modest, and several recommendations derive from guidance and consensus rather than randomized evidence, with practice continuing to evolve, as the changing role of lumbar puncture illustrates.

Notwithstanding these limitations, the approach is generalizable: soliciting questions from trainees and answering them against the current literature could be applied to other uveitic entities in which the gap between recognition and confident management is wide. The present work is offered both as a focused synthesis of ocular syphilis and as a template for such educational reviews.

## Conclusions

Ocular syphilis is an uncommon but sight-threatening manifestation of a resurgent infection, and prompt serologic testing with timely treatment remains the determinant of visual outcome. The questions raised by the responding residents point to a specific educational lesson: for these trainees, the challenge in ocular syphilis lay less in recognizing intraocular inflammation than in knowing what to do next, namely how to interpret serology, when to pursue systemic and cerebrospinal fluid evaluation, how to treat, and how to monitor the response. A question-driven review, by targeting exactly these points of uncertainty, offers a practical educational model that could be extended to other uveitic entities.
